# A lipidomic and flavoromic approach to map the lipid profile and related volatile flavor compounds in the fresh breast meat of chickens fed curcumin

**DOI:** 10.1016/j.fochx.2026.104156

**Published:** 2026-06-30

**Authors:** Sanjun Jin, Kaige Yang, Gaofeng Pan, Ping Wang, Chaoqi Liu, Xinxin Li, Qingqiang Yin, Juan Chang, Lijun Wang, Fushan Lu

**Affiliations:** a College of Animal Science and Technology, Henan Agricultural University, Zhengzhou 450046, China; b Henan Puai Feed Co. Ltd., Zhoukou 466000, PR China

**Keywords:** Chicken breast muscle, Dietary curcumin, Lipid metabolism, Volatile flavor compounds, Flavor characteristics

## Abstract

This study utilized multi-omics techniques to thoroughly characterize the volatile flavor compounds (VOCs) and lipids present in the breast muscle of chickens. These chickens were fed diets supplemented with 500 mg/kg curcumin (CUR) for 42 consecutive days. The findings revealed a significantly lower malondialdehyde (MDA) content in the chicken breast muscle of the CUR group compared to lower than that in the CON group (0.37 vs. 0.50; *P*<0.05), indicating reduced lipid oxidation. However, no significant difference in IMF content was observed between the two groups (*P* = 0.63). Furthermore, the flavor profiles exhibited significant variations between the groups, particularly in attributes such as green, fruity, fresh, fatty, herbal, and floral notes. Notably, VOCs like diphenyl sulfone and heptanal were identified as compounds associated with undesirable off-flavors. The lipid composition also showed significant differences between the CUR and CON groups, directly influencing the VOC profiles. The CUR group demonstrated reduced lipid oxidation and distinct volatile profiles, specifically with lower levels of the lipid oxidation-related VOCs diphenyl sulfone and heptanal. Collectively, this research establishes a theoretical foundation for modulating regulating the flavor-related volatile flavor profile of chicken meat through dietary CUR supplementation. This approach holds promise for the development of high-quality poultry products with enhanced flavor stability.

## Highlights

• CUR decreased MDA accumulation in chicken breast muscle.
• Predicted flavor attributes (green, fruity, fatty etc.) of chicken breast were altered by CUR treatment.
• CUR induced remarkable changes in lipid composition and volatile flavor compounds.
• CUR reduced the concentrations of diphenyl sulfone and heptanal derived from lipid oxidation.

## 1 Introduction

Chicken stands out as one of the globe's most extensively consumed sources of animal protein sources (Smiecinska et al., 2024). Its growing popularity stems from a combination of favorable nutritional attributes, palatable taste, and convenient processing and storage. On a global scale, poultry plays a crucial role in delivering high-quality animal protein, significantly bolstering food security and nutrition. Projections from the FAO anticipate that global poultry production will reach 181 million tons by 2050 (https://ourworldindata.org/grapher/global-meat-projections-to-2050). The sustainable development of the poultry industry hinges on continuous improvements in meat quality, a direct determinant of consumer acceptance and market competitiveness. Flavor, a primary indicator of meat sensory quality is crucial for repeat purchases (Lee et al., 2024). Meat flavor originates from volatile compounds produced through various biochemical pathways, including lipid oxidation, Maillard reactions, and the degradation of proteins and nucleotides (Bleicher et al., 2022). While research has elucidated many aspects of meat taste, certain key mechanisms remain undefined.

The distinctive savory notes, commonly referred to as “meaty” flavors, predominantly develop and intensify during the cooking process (Bassam et al., 2022). Raw meat, in contrast, possesses a subtle taste, often characterized by bloody or metallic undertones attributed to heme proteins. Despite its mild raw taste, meat is rich in precursors essential for developing these complex flavors upon cooking. Within connective tissues, the volatile compound profile, influenced by both inherent biochemistry and post-moratem reactions, is a critical contributor to overall meat flavor. These compounds undergo further transformation during storage and cooking.

Chicken meat is particularly vulnerable to lipid-protein oxidation due to its high concentration of pro-oxidative catalysts, such as myoglobin, iron, and unsaturated fatty acids. Lipid oxidation is a cascade reaction where unsaturated fatty acids react with molecular oxygen through free radical pathways, yielding primary oxidation products like hydroperoxides. These hydroperoxides, although odorless, are unstable intermediates that readily decompose into secondary metabolites such as aldehydes and alcohols. The impact of these secondary metabolites on meat off-odors depends on their concentration and corresponding detection thresholds (Jung et al., 2025). Such off-odors compromise sensory quality and consumer acceptance, thereby limiting market growth and innovation in chicken product development, Consequently, enhancing flavor and controlling off- flavors are urgent priorities for the industry.

Key biochemical pathways, such as lipid oxidation, the Strecker Reaction, the Maillard Reaction, and thiamin degradation, transform flavor precursors into volatile flavor compounds (VOCs). The predominant VOCs produced aldehydes, esters, ketones, hydrocarbons, furans, and alcohols (Jung et al., 2025). Flavor precursors are categorized into water-soluble compounds (including free amino acids, reducing sugars, peptides, thiamine, and organic acids) and fat-soluble compounds (mainly lipids and their derivatives) (Aaslyng & Meinert, 2017; Khan et al., 2015). Lipids are crucial substrates for flavor development and significantly shape the volatile compound profile during cooking and storage. They play a critical role in determining meat flavor by modulating the volatile compounds produced during processing (Khan et al., 2015). However, lipids abundant in unsaturated fatty acids are highly susceptible to oxidation during the post-mortem conversion of muscle into meat, which severely compromises flavor stability. The metabolic changes that occur after slaughter initiate both lipid and protein oxidation, resulting in rancidity and a decline in quality. Dietary strategies that boost antioxidant defenses can counteract these oxidation pathways, thereby maintaining meat quality and stability. Among various options, plant-derived polyphenols have gained recognition as promising natural feed additives due to their diverse biological properties. For example, Barbol and Alsayeqh (2024) demonstrated that polyphenols act as effective multifunctional feed additives, exhibiting potent antibacterial and antioxidant properties against *Campylobacter jejuni* in chickens. Rojas and Brewer (2008) demonstrated that a polyphenol-rich grape seed extract effectively inhibited lipid oxidation and improved the sensory attributes of beef and pork during processing. These findings suggest that polyphenols, owing to their potent antioxidant capacity, can be utilized to mitigate lipid oxidation and reduce the formation of off-flavor compounds in livestock meat, a conclusion further supported by existing research (Tahir et al., 2023; Papuc et al., 2017; Sohaib et al., 2017).

Curcumin (CUR), a natural polyphenol extracted from *Curcuma longa L*., is recognized for its affordability, safety, and widespread availability. It exhibits a diverse array of biological functions, encompassing antioxidant, anti-inflammatory, and antitumor activities, alongside its role in safeguarding mitochondrial function and signaling pathways (Benzer et al., 2018; Wang et al., 2018). CUR contributes to maintaining cell membrane stability and impeding lipid and /protein oxidation (Khan et al., 2012). Furthermore, in chickens, it has been shown to enhance meat quality and extend the shelf life of thigh meat by bolstering antioxidant capacity and suppressing lipid oxidation in chickens (Daneshyar, 2012). Our prior research established that an optimal dietary supplementation of CUR in poultry, at the levels between 400 and 500 mg/kg, effectively reduces lipid and protein oxidation, while simultaneously improving the structural stability of meat proteins during storage (Jin et al., 2020). Nevertheless, the majority of current research primarily investigates CUR's impact on growth performance, antioxidant status, and conventional meat quality attributes. The comprehensive effects of CUR on the comprehensive volatile flavor compounds and lipid profile composition of chicken breast muscle, particularly the intricate interplay among lipid metabolism, lipid oxidation, and flavor development, largely remain unexplored.

Based on the research background and identified knowledge gaps, this study hypothesized that dietary curcumin supplementation could enhance muscle flavor characteristics and reduce the concentration of undesirable off-flavor compounds. This improvement in flavor is posited to occur through the reshaping of lipid molecular profiles and the inhibition of lipid oxidation. Employing a combined approach of flavoromics and lipidomics, we analyzed variations in volatile flavor components while concurrently determining MDA content, IMF level, and overall lipid molecular characteristics in chicken breast muscle. Furthermore, we elucidated the intrinsic connection between flavor alterations and changes in lipid metabolism. This research clarifies the mechanism by which curcumin regulates chicken flavor, thereby providing a theoretical foundation for its application as a natural feed additive to improve meat flavor quality and facilitate the development of high-quality poultry products.

## 2 Materials and methods

### 2.1 Ethical approval

The experimental protocols were approved by the Institutional Animal Care and Use Committee (IACUC) of Henan Agricultural University (Approval No. 11–0099-2023).All chicken experiments were conducted strictly following the Guide for the Care and Use of Laboratory Animals published by the National Institutes of Health (NIH, USA), as well as the Guidelines for the Humane Killing of Experimental Animals (T/CALAS 31–2017) issued by the Chinese Association for Laboratory Animal Science.

### 2.2 Materials

Curcumin (CAS. 458–37-7, purity: 98.85%) was purchased from the Nanjing Jingzhu Bio-Technology Co., Ltd. (Nanjing, China). The batch number is JZ23061008, and the purity was confirmed by high-performance liquid chromatography (HPLC) according to the manufacturer's certificate of analysis.

### 2.3 Animals and treatments

A total of 120 healthy 817 chicken (males, 1-day-old) bought from Tongxu Poultry Breeding Farm (Henan Province, China) were used in a 42-day experiment. Chickens were randomly allocated into two groups following a completely randomized design, with 6 replicate cages per group and 10 chicken per cage: (I) the CON group, fed a basal diet alone; (II) the CUR group, fed a basal diet supplemented with 500 mg/kg curcumin for 42 d, as previously described (Jin et al., 2020). Curcumin was added to feed using a feed mixer according to the manufacturer's protocol. The basal diet (Table S1) in this experiment was formulated according to the National Research Council (Dale & N., 1994) and the Chinese chicken feeding standards (NY/T 823–2020-Performance Terminology and Measurements for Poultry). Chickens had ad libitum access to feed and water. All other management followed standard commercial chicken protocols.

### 2.4 Sample collection

On day 42, following a 12-h overnight fast, one chicken of similar body weight was randomly selected from each replicate cage (six birds per treatment group). The selection criteria included consistent growth performance, similar body weight, good health and stable physiological status of the broilers. Prior to dissection, the selected chickens were electrically stunned via a water bath stunner, then fully exsanguinated by cutting the carotid artery and jugular vein to achieve humane euthanasia. Breast muscle tissues were collected immediately afterwards.

On day 42, after a 12 h overnight feed withdrawal, one chicken with uniform body weight was randomly picked from each replicate cage (six birds per treatment). The selection criteria included consistent growth performance, similar body weight, good health and stable physiological status of the broilers. Prior to dissection, the selected chickens were electrically stunned via a water bath stunner, then fully exsanguinated by cutting the carotid artery and jugular vein to achieve humane euthanasia. Breast muscle tissues were collected immediately afterwards.

To investigate potential effects on subsequent measurements of MDA, IMF, and VOCs, chicken breast muscle samples were sealed in polyethylene bags. This ensured uniform effects on volatile compound profiles between the two groups. The samples were then incubated at 4 °C for 24 h to mimic post-mortem maturation prior to further analysis, a standard procedure in animal meat aging, prior to further analysis (Ruan et al., 2024; Dong et al., 2026).

### 2.5 MDA and IMF content

Chicken breast muscle samples were homogenized in prechilled 0.9% physiological saline solution (NO. ST341, Beyotime, Nanjing, China). A 10% (*w*/*v*) homogenate was produced and then centrifuged at 4 °C (3,000 ×g, 10 min). The resulting supernatants were subsequently analyzed for malondialdehyde (MDA) content using commercial thiobarbituric acid (TBA) kits (NO. A003–1-2, Nanjing Jiancheng Bioengineering Institute, China), following to the manufacturer's protocols. To quantify MDA, a calibration standard curve was established using 1,1,3,3-tetraethoxypropane (TEP) across a concentration range of 0–10 nmol/mL. The assay demonstrated strong precision, with an intra-assay CV of 3.2% and an inter-assay CV of 5.1%.

The IMF content of all chicken breast muscle samples was determined according to Chinese National Standard GB 5009.6–2016, “National Food Safety Standard - Determination of Fat in Foods. Briefly, freeze-dried chicken breast samples (1.0 g) were extracted with petroleum ether (60–90 °C) in a Soxhlet apparatus for 6 h. The IMF content was then reported as the percentage of fat weight relative to the dry sample weight.

### 2.6 Volatile compound headspace sampling for GC–MS analysis

#### 2.6.1 Extraction method

Solid-phase microextraction (SPME) is widely adopted technique for isolating volatile flavor compounds. It complements dynamic headspace analysis. The SPME extraction procedure followed established methodologies (Li et al., 2022; Sun et al., 2020) with few modifications. In this study, a SPME fiber (57330-U; Supelco) coated with divinylbenzene/carboxen/polydimethylsiloxane (DVB/CAR/PDMS; 50/30 μm × 1 cm) was selected for comprehensive profiling of chicken breast muscle volatiles, leveraging its superior adsorption capacity across broad polarity ranges.

A 2.00 g aliquot of meat homogenate was combined with 10 mL of a 6% sodium chloride solution in a 20 mL amber glass screw-capped headspace vial (22.5 × 75.5 mm, Thermo Fisher Scientific, Waltham, MA). Following this, 10.0 μL of an internal standard solution (n-Hexyl-d13 Alcohol, 1 mg/L in 50% ethanol) was precisely injected using a calibrated microsyringe. Method validation confirmed that the matrix recovery of n-Hexyl-d13 Alcohol in meat samples consistently fell between 86.4% and 92.7%, with a relative standard deviation (RSD) below 4.5%. This internal standard demonstrated robust stability and broad applicability for the semi-quantitative analysis of various volatile organic compounds in a meat matrix. The sealed vials were then thoroughly equilibrated in a temperature-controlled orbital shaker bath (80 °C) for 10 min. Prior to use, the DVB/CAR/PDMS SPME fiber was preconditioned at 250 °C for 10 min eliminate any residual contaminants before use. After sample equilibration, the purified fiber was exposed to the vial's headspace for 25 min at 80 °C to facilitate the adsorption of volatile compounds. These specific extraction parameters were chosen based on a previously validated study of meat volatiles that utilized an identical fiber type (Qi et al., 2025). Subsequently, the fiber was inserted into the GC injection port and thermally desorbed at 250 °C for 5 min.

The relative odor activity value (ROAV) was calculated from the semi-quantitative results of the volatile compounds to assess their individual contributions to the overall odor. The odor thresholds for all compounds were independently determined by an accredited third-party testing institution. In this context, ROAV served as a comparative reference index for relative comparisons between different groups under identical raw meat matrix conditions, rather than for an absolute evaluation of the odor activity in cooked meat.

#### 2.6.2 Gas chromatography coupled with time-of-flight mass spectrometry (GC × GC-TOF MS) analysis

Following thermal desorption of extracted volatile flavor compounds at 250 °C for 5 min in the injection port of a LECO Pegasus® 4D instrument (LECO, St. Joseph, MI, USA), which comprise an Agilent 8890 A GC (Agilent Technologies, Palo Alto, CA, USA) system equipped with a split/splitless injector, and dual stage cryogenic modulator (LECO) coupled with TOF MS detector (LECO).

A DB-Heavy Wax column (30 m × 250 μm I.D., 0.5 μm; Agilent, USA) was employed as the first-dimension column (^1^D), and an Rxi-5Sil MS column (2.0 m × 150 μm I.D., 0.15 μm; Restek, USA) served as the second-dimension column (^2^D). Ultra-high purity helium (>99.999%) carrier gas was maintained at a constant flow rate of 1.0 mL/min. The GC oven temperature program began with an initial hold at 50 °C for 2 min. Subsequently, the temperature was ramped at a rate of 5 °C/min ramp to 230 °C with a 5 min isothermal period. The secondary oven's temperature was maintained at an offset of +5 °C relative to the primary oven. The modulator operated with a + 15 °C differential above the secondary column temperature, executing 6.0-s cycles. The injector temperature was consistently maintained at 250 °C.

Flavor substances were analyzed using a LECO Pegasus BT 4D instrument. The transfer line temperature and TOF MS ion source temperatures were bouth set to 250 °C. Data were acquired at a frequency of 200 spectra per second. The mass spectrometer was operated in electron ionization (EI) mode with an electron energy of 70 eV, a mass-to-charge ratio (*m*/*z*) range of 35–550, and a detector voltage of 1960 V.

### 2.7 Untargeted lipidomic analysis

The untargeted lipidomic analysis was conducted by Suzhou Panomix Biomedical Tech Co. Ltd. (Suzhou, China) using a two-step methodology involving lipid extraction followed by liquid chromatograph-mass spectrometry (LC-MS) analysis. To uphold data integrity, procedural blanks and pooled quality control (QC) samples were concurrently prepared and analyzed throughout the experimental process. All samples were processed within a single analytical batch, thereby eliminating batch-to-batch variation without requiring subsequent batch correction. Universal lipid internal standards were employed for the relative quantification of various lipid classes, and method validation included the calculation of the limit of detection (LOD) and limit of quantification (LOQ).

#### 2.7.1 Lipid extraction

A 100 mg chicken breast muscle sample and two glass beads were placed in a centrifuge tube with two glass beads. Precisely 750 μL of pre-chilled (−20 °C) chloroform: methanol (2:1, *v*/v) was added. The mixture was vortexed in a high-throughput tissue homogenizer (SCIENTZ-48, Ningbo, China) at 50 Hz for 60 s, repeated twice. After incubating on ice for 40 min, 190 μL of ultrapure water was added, followed by vortexing for 30 s and incubation at −20 °C for 10 min. The sample was centrifuged at 13000 *g* for 5 min at room temperature. A 300 μL aliquot of the lower organic layer was transferred to a new centrifuge tube. Subsequently, 500 μL of chloroform: methanol (2:1, v/v) was added, and the mixture was vortexed for 30 s before centrifugation at 13000 *g* for 5 min at room temperature. A 400 μL aliquot of this newly formed lower organic layer was then combined with the previously obtained. The pooled organic extracts were subsequently dried under vacuum. The residue was dissolved in 200 μL isopropanol, filtered through a 0.22 μm membrane, and prepared for LC-MS analysis (Werner et al., 2018). Procedural blanks and pooled quality control (QC) samples were prepared synchronously throughout the extraction procedure. The pooled QC was prepared by mixing equal volumes of all individual samples and injected every ten samples to monitor instrument stability and analytical repeatability. All samples were analyzed in a single analytical batch to eliminate batch variation. Universal lipid internal standards were used for relative quantification. The limit of detection (LOD) and limit of quantification (LOQ) were determined for method validation. The LOD and LOQ values of the established method were calculated based on the signal-to-noise (S/N) ratio. LOD was defined as S/*N* ≥ 3, and LOQ was defined as S/*N* ≥ 10.

#### 2.7.2 Liquid chromatography-mass spectrometry (LC-MS) analysis

Chromatographic separation was achieved using an ACQUITY UPLC® BEH C18 column (2.1 × 100 mm, 1.7 μm; Waters) maintained at 50 °C. The autosampler temperature set at 8 °C. A binary mobile phase system was employed, consisting of A2 [acetonitrile/water (60:40, *v*/v) with 0.1% formic acid and 10 mM ammonium formate] and B2 [isopropanol/acetonitrile (90:10, v/v) with 0.1% formic acid and 10 mM ammonium formate], delivered at a flow rate of 0.25 mL/min. Following column equilibration, the flow rate was set at 0.25 mL/min, and each sample was injected in 2 μL.

A total running time of 28 min was applied for gradient elution, and the detailed elution procedure was clearly defined as follows: 70–57% A2 from 0 to 5 min; 57–50% A2 from 5 to 5.1 min; 50–30% A2 from 5.1 to 14 min; isocratic maintenance at 30% A2 from 14 to 14.1 min; 30–1% A2 from 14.1 to 21 min; isocratic maintenance at 1% A2 from 21 to 24 min; rapid linear increase from 1% to 70% A2 within 24.0–24.1 min; and continuous maintenance at 70% A2 for column re-equilibration during 24.1–28 min.

For ESI-MSn analysis, spray voltages were set at 3.5 kV in positive mode and 2.5 kV in negative mode. Sheath with sheath and auxiliary gases were maintained at 30 and 10 arbitrary units, respectively, with the capillary temperature was held at 325 °C. Full-scan MS data (*m*/*z* 150–2000) were acquired in the Orbitrap analyzer at 35,000 resolutions, with data-dependent MS/MS scans triggered using HCD fragmentation at 30 eV normalized collision energy. Dynamic exclusion was applied to simplify MS^2^ spectra (Dasilva et al., 2019).

### 2.8 Data analysis

The relevant results (MDA and IMF) were denoted as the Mean ± SD. Statistical analysis was performed using Student's *t*-tests with SPSS (Version 28.0, Chicago, IL, USA). To facilitate comparative analysis of flavor compounds with significantly varying concentrations, raw chromatographic data were normalized using the total peak area method. Flavor-related constituents were subsequently annotated by referencing the NIST 2020 mass spectral library within the ChromaTOF software.

The detected flavor compounds were classified and quantitatively analyzed across chemical classes using the PubChem database (https://pubchem.ncbi.nlm.nih.gov/) integrated with ClassyFire ontology-based chemoinformatics software. This approach enabled the statistical determination of compound distribution and relative abundances within each chemical category.

To enable comparative analysis of lipids, particularly those with significant concentration variations, raw data were first converted to a compatible format. Subsequently, these data were imported into LipidSearch ver 4.0 for a comprehensive preprocessing workflow, encompassing data collection through to alignment. Putative lipid annotations were then assigned by comparing experimental precursor ion *m*/*z* values and product ion patterns against the LipidSearch database. Finally, batch effects were mitigated through normalization based on the total peak area.

Orthogonal partial least squares-discriminant analysis (OPLS-DA) was implemented for multivariate statistical modeling of inter-group discriminative patterns. Inter-group differential significance was assessed using Student's *t*-tests and one-way ANOVA. Prior to parametric analysis, all metabolite data were log₂-transformed for normal distribution normalization, and missing values were imputed with half of the minimum detectable value. False discovery rate (FDR) correction was applied to mitigate false positive errors in high-dimensional omics data. Differential metabolites were screened based on the criteria of VIP >1 and FDR < 0.05.

## 3 Results and discussion

### 3.1 MDA and IMF contents in the chicken breast muscle

MDA, a product of lipid peroxidation, serves as a crucial biomarker for assessing oxidative damage in meat, with elevated concentrations correlating strongly with diminished meat quality. IMF content influences fatty acid composition, the generation of volatile compounds during cooking, and the kinetics of pre- and post-swallow volatile release in cooked meat. Consequently, comprehensive lipid profiling is imperative to elucidate the mechanisms underpinning meat flavor development. Data presented in Table 1 indicate that dietary CUR supplementation significantly reduced MDA content in chicken breast muscle compared to that in the CON group (*P* < 0.05), while no significant difference in IMF content was observed. This study demonstrated that dietary CUR supplementation inhibited MDA accumulation in meat. This finding aligns with previous research indicating that antioxidant-enriched diets enhance antioxidant deposition in chicken breast muscle, thereby improving redness by suppressing lipid and protein oxidation (Adeyemi, 2021). Similarly, Bashir et al. (2024) showed that *Moringa oleifera* leaf extract prevented tissue injury in rats by improving hepatic cellular integrity, regulating liver protein and enzyme levels, and reducing TBARS content through the activation of antioxidant enzymes. Nastoh et al. (2025) also reported that *Moringa oleifera* leaf extract enhanced the antioxidant capacity in broilers. Furthermore, Zhang et al. (2025) found that dietary antioxidant supplementation improved meat quality by reducing protein oxidation. Given that IMF is rich in lipids, which are susceptible to the oxidation and degradation and directly influence flavor compound composition, the lipids in the samples were further analyzed to investigate this mechanistic relationship.

**Table 1:** Effects of different dietary CUR supplementation on MDA and IMF contents of chicken breast muscle.

Items	Groups		*P*-values
	CON	CUR	
MDA (nmol/mg prot)	0.50 ± 0.10 ^a^	0.37 ± 0.04 ^b^	0.04
IMF (%)	1.62 ± 0.10	1.66 ± 0.11	0.63

Note: Data are expressed as mean ± SD.

Malondialdehyde: MDA; Intramuscular fat: IMF.

### 3.2 Difference analysis of chicken breast muscle volatile flavor compounds

#### 3.2.1 Types of volatile flavor compounds and overall analysis for sensory flavor characteristics

**Fig. 1:**
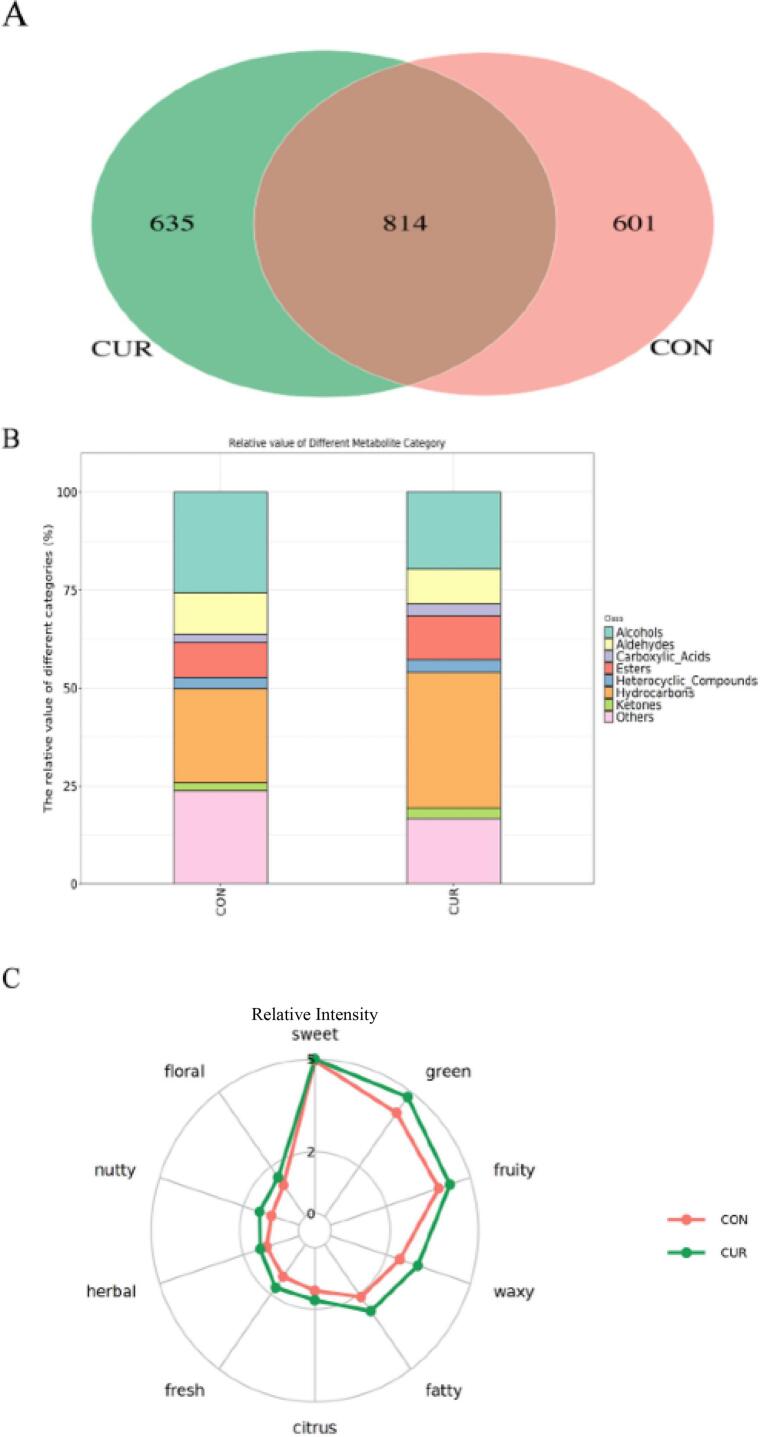
Mass spectrum from GC × GC-TOF MS analysis of different chicken breast muscle sample. (A) VOCs (volatile flavor compounds) identified in samples using GC × GC-TOF MS. (B) Relative value of different metabolite category. (C) Radar plot of predicted flavor attributes in chicken meat from CON and CUR groups. The radial axis represents the relative intensity of each flavor attribute, which was derived from volatile compound profiles and matched with odor descriptors using the FlavorDB database. No human sensory panel evaluation was conducted.

VOCs from the CON and CUR groups were meticulously analyzed using (comprehensive two-dimensional gas chromatography coupled with time-of-flight mass spectrometry) GC × GC-TOF MS. Following rigorous compound annotation and the elimination of contaminants, a substantial total of 1449 volatile flavor compounds were identified and quantified (Fig. 1A and Table S2). These compounds were categorized into eight distinct classes: alcohols, aldehydes, carboxylic_acids, esters, heterocyclic_compounds, hydrocarbons, ketones, and other compounds (Fig. 1B and Table S3). A notable observation was the presence of 34 additional VOCs in the chicken breast muscle from the CUR group compared to the CON group, signifying a greater diversity of VOCs in the CUR group. The identified VOC categories align with previous research by Jin et al. (2021), which also reported an increase in VOC diversity in duck breast muscle following curcumin supplementation. Further in-depth analysis in this study revealed clear differences in the relative intensities of the primary VOC classes between the two groups (Table S3). Specifically, the CUR group exhibited higher relative intensities for carboxylic _ acids (3.06 vs 2.15), esters (11.14 vs 8.83), heterocyclic _ compounds (3.39 vs 2.86), hydrocarbons (34.48 vs 24.00) and ketones (2.72 vs 2.10) when compared to the CON group. Conversely, the relative intensities of alcohols (19.64 vs 25.63), aldehydes (8.83 vs 10.64), and other compounds (16.75 vs 23.79) were lower in the CUR group. The extensive array of VOCs detected in the chicken breast muscle likely contributes to its intricate and multifaceted flavor profile.

The overall flavor features were further predicted based on volatile profiles via the FlavorDB database (Fig. 1C). Flavor DB facilitated the prediction of sensory characteristics for VOCs in chicken breast muscle from both the CUR and CON groups (Fig. 1C, Table S4). Table S4 provides the predicted odor profiles, matched against the database, for these chicken breast muscle specimens. Although the CUR and CON chicken breast muscle shared similar overarching sensory profiles, CUR samples consistently showed higher predicted relative intensities across several key attributes, including green, fruity, waxy, fatty, citrus, fresh, nutty, and floral notes. This difference in predicted odor attributes correlates with variations in their volatile flavor compounds. Such distinctions in volatile flavor compounds between the samples are primarily linked to dietary polyphenol supplementation (Jin et al., 2021a; Jin et al., 2021b). Polyphenols, well-known for their strong antioxidant capabilities, reduce lipid oxidation in raw meat, thus presenting a method to modify volatile flavor profiles and their corresponding predicted odor characteristics.

#### 3.2.2 Analysis of differential volatile flavor compounds

**Fig. 2:**
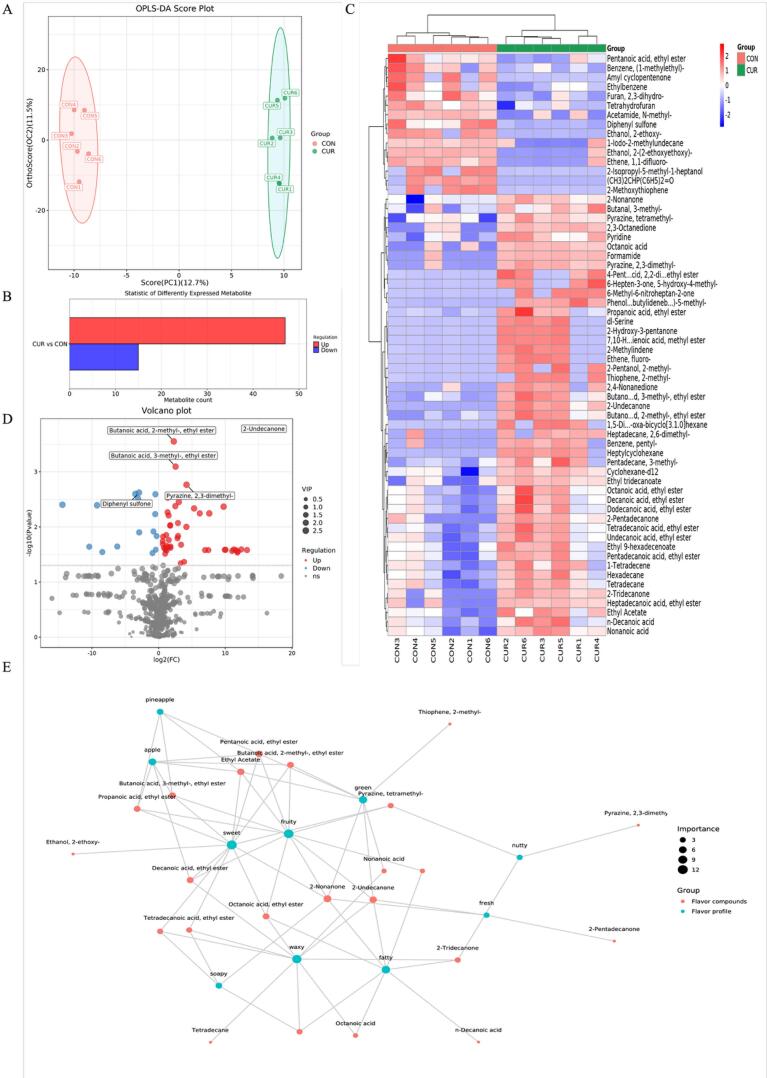
GC × GC-TOF MS analysis of different chicken breast muscle samples. (A) OPLS-DA analysis of the chicken breast muscle sample between CON and CUR groups. OPLS-DA model parameters: (R^2^Y = 1.00), (Q^2^ = 0.34); permutation validation is shown in Supplementary Fig. S1 to verify no overfitting. (B) Statistic of different expressed metabolite for chicken breast muscle. (C) Volcano plot of differentially abundant VOCs in the chicken breast muscle. (D) Heatmap of annotated differential VOCs expression in the chicken breast muscle between the CON and CUR groups with values normalized (*P* < 0.05) (Relative content is represented by colour intensity: red indicates higher content, while blue indicates lower content. Columns represent samples, and rows represent substances. The cluster tree on the left side shows hierarchical clustering of the substances.). (E) The interaction map of VOCs and each flavor indicating the degree of contribution of VOCs to the flavor (Green circles represent sensory features, and red circles represent flavor compounds. For green circles: The larger the circle, the more flavor compounds it connects to, and the more important the sensory feature is. For red circles: The larger the circle, the more sensory features it associates with, and the more important the flavor compound is). (For interpretation of the references to colour in this figure legend, the reader is referred to the web version of this article.)

As presented in Fig. 2A, the OPLS-DA model effectively distinguished between the CON and CUR groups. To identify the specific molecules responsible for the off-odor, 62 differential VOCs were selected based on their variable importance in projection thresholds (VIP) > 1.0 and adjusted *P*-value <0.05 (Fig. 2B). CUR showed significant up-regulation (*P* < 0.05) of 39 VOCs and down-regulation (*P* < 0.05) of 14 compared to CON (Table 2 and Table S9). These differential VOCs created distinct sensory profiles between the CUR and CON chicken breast muscle samples. The differential VOCs observed between CUR and CON chicken breast muscle primarily fell into 13 classes: 2 Alcohols, 1 Aldehydes, 5 Benzenoids, 16 Esters, 7 Hydrocarbons, 6 Ketones, 3 Lipids and lipid-like molecules, 1 Organic 1,3-dipolar compounds, 1 Organic acids and derivatives, 2 Organic oxygen compounds, 1 Organohalogen compounds, 7 Organoheterocyclic compounds and 1 other.

**Table 2:** Variations in differential volatile flavor compounds in chicken breast muscle affected by CUR (CUR vs CON).

Count	Name	Class	CAS	Formula	CUR_Mean	CON_Mean	FC	*P.*value	VIP	Regulation
1	2-Isopropyl-5-methyl-1-heptanol	Alcohols	91,337–07-4	C11H24O	0.00	0.02	0.00	0.02	1.96	Down
2	2-Pentanol, 2-methyl-	Alcohols	590–36-3	C6H14O	0.04	0.00	2073.26	0.03	1.90	Up
3	Butanal, 3-methyl-	Aldehydes	590–86-3	C5H10O	1.03	0.66	1.55	0.02	1.80	Up
4	Benzene, (1-methylethyl)-	Benzenoids	98–82-8	C9H12	0.01	0.01	0.74	0.03	1.76	Down
5	(CH3)2CHP(C6H5)2 = O	Benzenoids	2959-75-3	C15H17OP	0.00	0.36	0.00	0.00	2.32	Down
6	Ethylbenzene	Benzenoids	100–41-4	C8H10	0.19	0.24	0.81	0.01	1.86	Down
7	Benzene, pentyl-	Benzenoids	538–68-1	C11H16	0.01	0.00	7.06	0.02	1.85	Up
8	Diphenyl sulfone	Benzenoids	127–63-9	C12H10O2S	0.10	0.78	0.13	0.00	2.37	Down
9	7,10-Hexadecadienoic acid, methyl ester	Esters	16,106–03-9	C17H30O2	0.04	0.00	2462.86	0.03	1.94	Up
10	Ethyl 9-hexadecenoate	Esters	54,546–22-4	C18H34O2	1.52	0.66	2.31	0.03	1.75	Up
11	Butanoic acid, 3-methyl-, ethyl ester	Esters	108–64-5	C7H14O2	0.10	0.02	5.82	0.00	2.27	Up
12	Propanoic acid, ethyl ester	Esters	105–37-3	C5H10O2	0.30	0.14	2.08	0.02	1.93	Up
13	Ethyl Acetate	Esters	141–78-6	C4H8O2	13.97	4.19	3.33	0.01	1.94	Up
14	Pentanoic acid, ethyl ester	Esters	539–82-2	C7H14O2	0.02	0.03	0.59	0.01	1.90	Down
15	Undecanoic acid, ethyl ester	Esters	627–90-7	C13H26O2	0.03	0.01	2.20	0.01	1.89	Up
16	Pentadecanoic acid, ethyl ester	Esters	41,114–00-5	C17H34O2	0.21	0.11	1.97	0.03	1.73	Up
17	Tetradecanoic acid, ethyl ester	Esters	124–06-1	C16H32O2	4.34	0.77	5.66	0.00	2.09	Up
18	Ethyl tridecanoate	Esters	28,267–29-0	C15H30O2	0.05	0.01	10.35	0.02	1.95	Up
19	Heptadecanoic acid, ethyl ester	Esters	14,010–23-2	C19H38O2	0.03	0.01	3.41	0.02	1.93	Up
20	Dodecanoic acid, ethyl ester	Esters	106–33-2	C14H28O2	1.66	0.48	3.47	0.02	1.81	Up
21	Butanoic acid, 2-methyl-, ethyl ester	Esters	7452-79-1	C7H14O2	0.15	0.03	4.85	0.00	2.36	Up
22	Decanoic acid, ethyl ester	Esters	110–38-3	C12H24O2	1.02	0.35	2.88	0.02	1.86	Up
23	Ethanol, 2-(2-ethoxyethoxy)-	Ethers	111–90-0	C6H14O3	0.00	0.02	0.09	0.00	2.37	Down
24	Ethanol, 2-ethoxy-	Ethers	110–80-5	C4H10O2	0.00	0.01	0.00	0.00	2.32	Down
25	1-Tetradecene	Hydrocarbons	1120-36-1	C14H28	0.06	0.04	1.73	0.02	1.77	Up
26	Hexadecane	Hydrocarbons	544–76-3	C16H34	1.40	0.88	1.60	0.01	1.88	Up
27	Tetradecane	Hydrocarbons	629–59-4	C14H30	2.46	1.61	1.53	0.02	1.84	Up
28	Cyclohexane‑*d*_12_	Hydrocarbons	1735-17-7	C6D12	112.54	56.15	2.00	0.03	1.90	Up
29	Heptylcyclohexane	Hydrocarbons	5617-41-4	C13H26	0.01	0.00	859.67	0.00	2.30	Up
30	Pentadecane, 3-methyl-	Hydrocarbons	2882-96-4	C16H34	0.17	0.06	2.83	0.01	2.02	Up
31	Heptadecane, 2,6-dimethyl-	Hydrocarbons	54,105–67-8	C19H40	0.21	0.08	2.68	0.02	1.78	Up
32	Amyl cyclopentenone	Ketones	4819-67-4	C10H18O	0.00	0.01	0.00	0.03	1.90	Down
33	2-Pentadecanone	Ketones	2345-28-0	C15H30O	0.03	0.00	7.49	0.01	1.96	Up
34	2-Tridecanone	Ketones	593–08-8	C13H26O	0.24	0.01	39.98	0.00	2.21	Up
35	2-Nonanone	Ketones	821–55-6	C9H18O	0.45	0.03	14.27	0.04	1.71	Up
36	2,4-Nonanedione	Ketones	6175-23-1	C9H16O2	0.05	0.00	71.89	0.01	2.03	Up
37	2,3-Octanedione	Ketones	585–25-1	C8H14O2	0.96	0.05	17.99	0.01	2.13	Up
38	n-Decanoic acid	Lipids and lipid-like molecules	334–48-5	C10H20O2	0.04	0.02	2.29	0.02	1.80	Up
39	Octanoic acid	Lipids and lipid-like molecules	124–07-2	C8H16O2	0.15	0.02	7.56	0.01	2.05	Up
40	Nonanoic acid	Lipids and lipid-like molecules	112–05-0	C9H18O2	0.21	0.08	2.52	0.01	2.03	Up
41	6-Methyl-6-nitroheptan-2-one	Organic 1,3-dipolar compounds	142,963–25-5	C8H15NO3	0.05	0.00	3133.70	0.03	1.92	Up
42	Acetamide, *N*-methyl-	Organic acids and derivatives	79–16-3	C3H7NO	0.01	0.04	0.13	0.01	2.08	Down
43	Ethene, 1,1-difluoro-	Organic oxygen compounds	75–38-7	C2H2F2	0.01	0.09	0.12	0.00	2.27	Down
44	2-Hydroxy-3-pentanone	Organic oxygen compounds	5704-20-1	C5H10O2	0.09	0.00	5446.61	0.03	1.94	Up
45	1-Iodo-2-methylundecane	Organohalogen compounds	73,105–67-6	C12H25I	0.05	0.10	0.53	0.03	1.87	Down
46	Pyridine	Organoheterocyclic compounds	110–86-1	C5H5N	0.53	0.20	2.58	0.00	2.12	Up
47	Tetrahydrofuran	Organoheterocyclic compounds	109–99-9	C4H8O	0.14	0.20	0.70	0.01	2.05	Down
48	Furan, 2,3-dihydro-	Organoheterocyclic compounds	1191-99-7	C4H6O	0.10	0.14	0.72	0.00	2.14	Down
49	Pyrazine, 2,3-dimethyl-	Organoheterocyclic compounds	5910-89-4	C6H8N2	0.05	0.00	18.25	0.00	2.37	Up
50	Pyrazine, tetramethyl-	Organoheterocyclic compounds	1124-11-4	C8H12N2	0.05	0.01	9.84	0.05	1.70	Up
51	1,5-Dimethyl-6-oxa-bicyclo [3.1.0] hexane	Organoheterocyclic compounds	82,461–31-2	C7H12O	0.02	0.00	1062.37	0.03	1.92	Up
52	Octanoic acid, ethyl ester	Organoheterocyclic compounds	106–32-1	C10H20O2	2.34	0.70	3.32	0.01	1.96	Up
53	Formamide		75–12-7	CH3NO	0.52	0.06	8.18	0.00	2.33	Up

CUR_Mean and CON_Mean are average means, *n* = 6. CON, chicken fed with standard diet; CUR, chickens fed with standard diet with 500 mg/kg CUR.

The regulation in the CUR group compared to the CON group. RT, retention time; FC, fold change; VIP, Variable importance in projection.

Hierarchical clustering analysis of the differential VOCs revealed significant differences between the CUR and CON groups (Fig. 2C). A volcano plot was generated to visualize the top 5 upregulated and downregulated volatile flavor compounds (Fig. 2D and Table 3). Four differential volatile flavor compounds were significantly elevated (*P* < 0.05) in CUR relative to CON: butanoic acid, 2-methyl- ethyl ester (Fruity, FlavorDB), 2-undecanone (Orange, Fresh, Green, FlavorDB), butanoic acid, 3-methyl-, ethyl ester (Fruity, Apple-Flavor), and pyrazine, 2,3-dimethyl (Toasted Bread, Fried Corn, Roasted Buns, Roasted Peanuts, FlavorDB). Conversely, diphenyl sulfone significantly decreased (*P* < 0.05). CUR supplementation improved meat flavor profiles while inhibiting the production of off-flavor compounds (e.g., diphenyl sulfone, FlavorDB). This aligns with Wu et al. (2024), who reported that polyphenols mitigate lipid oxidation in meat through potent antioxidant activity, thereby reducing lipid-derived off-flavors.

**Table 3:** ROAV in differential volatile flavor compounds in chicken breast muscle.

Name	CAS	Formula	CON_ROAV	CUR_ROAV	Odor Character
2-Hexen-1-ol, (*E*)-	928–95-0	C6H12O	0.00	0.00	Fruity, Fresh, Green
1-Octen-3-ol	3391-86-4	C8H16O	0.06	0.05	Mushroom
1-Heptanol	111–70-6	C7H16O	0.01	0.00	Grassy
1-Butanol, 3-methyl-	123–51-3	C5H12O	0.01	0.03	Sweet, Malty, Rancid, Rubber,
1-Propanol, 2-methyl-	78–83-1	C4H10O	0.00	0.00	Sweet, Fusel, Musty, Alcohol, Rubber, Latex
Ethanol	64–17-5	C2H6O	0.00	0.00	Disolvent, Ethanol
2-Propanol, 2-methyl-	75–65-0	C4H10O	0.00	0.00	Sweet Alcohol
1-Propanol	71–23-8	C3H8O	0.00	0.00	Fruity, Floral, Grassy
1-Hexanol, 2-ethyl-	104–76-7	C8H18O	21.25	24.40	Rose, Green
2,3-Butanediol	513–85-9	C4H10O2	0.01	0.21	Fruity, Onion
1-Octanol	111–87-5	C8H18O	0.29	0.24	Penetrating
1-Pentanol	71–41-0	C5H12O	0.04	0.03	
2-Propanol, 1-methoxy-	107–98-2	C4H10O2	0.00	0.00	Etherish, Ammonia
1-Hexanol	111–27-3	C6H14O	0.05	0.04	Green Grass, Plastic
1-Butanol	71–36-3	C4H10O	0.02	0.01	Sweet, Malty, Alcohol, Medicinal
2-Nonenal, (E)-	18,829–56-6	C9H16O	100.00	96.99	Fatty, Cucumber
2-Hexenal, (E)-	6728-26-3	C6H10O	0.00	0.00	Apple-Like, Planty Green, Stinkbug
2,4-Decadienal, (E,E)-	25,152–84-5	C10H16O	0.00	0.00	Dusty, Waxy, Oily, Soapy
2-Octenal, (E)-	2548-87-0	C8H14O	12.88	12.43	Nuts, Green, Fatty
Acetaldehyde	75–07-0	C2H4O	3.02	2.25	Pungent, Fruity, Suffocating, Fresh, Green
Propanal, 2-methyl-	78–84-2	C4H8O	0.29	0.25	Pungent
Octanal	124–13-0	C8H16O	0.12	0.09	Lemon, Citrus, Green Grass
Pentanal	110–62-3	C5H10O	0.97	0.78	Sickening, Rancid, Decayed
Nonanal	124–19-6	C9H18O	0.25	0.32	Aldehyde, Citrus, Orange Peel
Hexanal	66–25-1	C6H12O	0.06	0.04	Green Grass, Fruity
Heptanal	111–71-7	C7H14O	50.85	32.16	Citrus, Fatty, Rancid
Butanal	123–72-8	C4H8O	0.52	0.29	Pungent
Propanal	123–38-6	C3H6O	0.02	0.02	Fruity
Butanal, 3-methyl-	590–86-3	C5H10O	0.01	0.01	Floral, Fruity
Butanal, 2-methyl-	96–17-3	C5H10O	19.68	28.99	Cocoa, Almond
Benzene, (1-methylethyl)-	98–82-8	C9H12	0.00	0.00	Sharp
Aniline	62–53-3	C6H7N	0.00	0.00	Pungent, Oily, Empyreumatic
Pentaborane(9)	19,624–22-7	B5H9	0.00	0.00	Pungent
Benzene, chloro-	108–90-7	C6H5Cl	0.00	0.00	Almond-Like, Shoepolish
p-Cresol	106–44-5	C7H8O	0.00	0.00	
Benzene	71–43-2	C6H6	0.00	0.00	Aromatic, Sweet, Solvent, Empyreumatic
Benzene, 1,4-dichloro-	106–46-7	C6H4Cl2	0.00	0.00	Camphor, Mothballs
2-Propenoic acid, 2-methyl-	79–41-4	C4H6O2	0.00	0.00	Pungent
2-Propenoic acid, ethyl ester	140–88-5	C5H8O2	0.87	0.23	Sweet, Ester, Plastic, Alcohol, Sharp, Ammoniacal
2-Propenoic acid, butyl ester	141–32-2	C7H12O2	0.10	0.03	Sweet, Rancid, Plastic
Butanoic acid, 3-methyl-, ethyl ester	108–64-5	C7H14O2	0.00	0.00	Fruity, Apple-Flavor
Propanoic acid, ethyl ester	105–37-3	C5H10O2	0.00	0.00	Ruity, Banana-Flavor
Ethyl Acetate	141–78-6	C4H8O2	0.01	0.03	Fruity, Sweet, Fingernail Polish, Etherous
Hexadecanoic acid, ethyl ester	628–97-7	C18H36O2	0.70	0.91	Wax
Propanoic acid, 2-methyl-, ethyl ester	97–62-1	C6H12O2	0.00	0.00	Fruity, Strawberry
Pentanoic acid, ethyl ester	539–82-2	C7H14O2	0.00	0.00	Fruity, Apple-Flavor
Tetradecanoic acid, ethyl ester	124–06-1	C16H32O2	0.00	0.00	Sweet, Waxy
Dodecanoic acid, ethyl ester	106–33-2	C14H28O2	0.00	0.00	Sweet, Waxy, Floral
Propanoic acid, 2-hydroxy-, ethyl ester	97–64-3	C5H10O3	0.00	0.00	Fruity, Slightly Fatty Flavor
Nonanoic acid, ethyl ester	123–29-5	C11H22O2	0.00	0.00	Floral, Fruity
Ethyl formate	109–94-4	C3H6O2	0.00	0.00	Aromatic
Hexanoic acid, ethyl ester	123–66-0	C8H16O2	0.00	0.00	Fruity, Green Apple
Butanoic acid, 2-methyl-, ethyl ester	7452-79-1	C7H14O2	0.00	0.01	Fruity
Butanedioic acid, diethyl ester	123–25-1	C8H14O4	0.00	0.00	Fruity, Sweet
Decanoic acid, ethyl ester	110–38-3	C12H24O2	0.00	0.00	Brandy
Heptanoic acid, ethyl ester	106–30-9	C9H18O2	0.00	0.00	Fruity
Butanoic acid, ethyl ester	105–54-4	C6H12O2	0.00	0.00	Fruity
Ethane, 1,1-diethoxy-	105–57-7	C6H14O2	0.00	0.00	Fruity
Dimethyl sulfide	75–18-3	C2H6S	0.10	0.09	Disagreeable, Asparagus, Putrid
Ethanol, 2-ethoxy-	110–80-5	C4H10O2	0.00	0.00	Sweet, Musty
Furan, 2-methyl-5-(methylthio)-	13,678–59-6	C6H8OS	0.00	0.00	Mustard, Onion
Ethanol, 2-butoxy-	111–76-2	C6H14O2	0.00	0.00	Sweet, Ester, Musty
Cyclohexene	110–83-8	C6H10	0.00	0.00	Sweet
gamma-Terpinene	99–85-4	C10H16	0.00	0.00	Oily, Smoky
1-Decene	872–05-9	C10H20	0.00	0.00	Pleasant
Octane	111–65-9	C8H18	0.00	0.00	Gasoline, Oil
5-Hepten-2-one, 6-methyl-	110–93-0	C8H14O	0.00	0.00	Herby, Green, Citrus, Musty, Lemongrass
1-Octen-3-one	4312-99-6	C8H14O	4.85	7.25	Mushroom-Like
2-Butanone	78–93-3	C4H8O	0.01	0.00	Sweet, Sharp
Acetone	67–64-1	C3H6O	0.00	0.00	Sweet, Fruity, Etherous
3-Heptanone, 5-methyl-	541–85-5	C8H16O	0.00	0.00	Solvent, Sharp
2-Acetyl-5-methylfuran	1193-79-9	C7H8O2	0.00	0.00	Biscuits, Toasted Almonds, Soap
2-Undecanone	112–12-9	C11H22O	0.00	4.51	Orange, Fresh, Green
2-Nonanone	821–55-6	C9H18O	0.00	0.00	Fruity, Floral, Fatty
2-Heptanone	110–43-0	C7H14O	0.08	0.07	Sweet, Mushroom
2,3-Butanedione	431–03-8	C4H6O2	22.36	13.43	Pleasant, Buttery
Myrcene	123–35-3	C10H16	0.00	0.00	Geranium
n-Decanoic acid	334–48-5	C10H20O2	0.00	0.00	Fatty, Rancid, Soap
p-Cymene	99–87-6	C10H14	0.00	0.00	
a-Terpineol	98–55-5	C10H18O	0.02	0.01	Piney, Iris, Teil
Dodecanoic acid	143–07-7	C12H24O2	0.00	0.00	Greasy, Slightly, Pine, Wood
Octanoic acid	124–07-2	C8H16O2	0.00	0.00	Rancid, Cheese, Fatty Acid
Nonanoic acid	112–05-0	C9H18O2	0.00	0.00	Fat Smell
1-Butanamine, N-butyl-	111–92-2	C8H19N	0.00	0.00	Amine
2-Propenal	107–02-8	C3H4O	0.04	0.01	Pungent
Ethane, 1,2-dichloro-	107–06-2	C2H4Cl2	0.00	0.00	Sweet
Thiophene	110–02-1	C4H4S	0.00	0.00	Garlic, Alliaceous
Pyridine	110–86-1	C5H5N	0.00	0.01	Burnt, Pungent, Nauseating
Thiophene, 3-methyl-	616–44-4	C5H6S	0.00	0.00	Plastic, Sulfurous
Tetrahydrofuran	109–99-9	C4H8O	0.00	0.00	Ether
Thiazole	288–47-1	C3H3NS	0.00	0.00	Nut, Sulfur, Stink
Furan, 2-ethyl-	3208-16-0	C6H8O	0.00	0.00	Burnt, Sweet, Coffee-Like
Furan, 2-pentyl-	3777-69-3	C9H14O	28.94	25.33	Green Beans, Vegetable
Pyrazine, methyl-	109–08-0	C5H6N2	0.00	0.00	Toasted Bread, Roasted Almonds, Fried Peanuts
Pyridine, 2-methyl-	109–06-8	C6H7N	0.00	0.00	Strong, Unpleasant
Pyrazine, 2,5-dimethyl-	123–32-0	C6H8N2	0.00	0.00	Green Grass, Fried Bean Spice
Pyrazine, 2,3-dimethyl-	5910-89-4	C6H8N2	0.00	0.00	Toasted Bread, Fried Corn, Roasted Buns, Roasted Peanuts
Pyrazine, trimethyl-	14,667–55-1	C7H10N2	0.03	0.24	Roasted Nuts, Cocoa, Peanuts
Pyrazine, tetramethyl-	1124-11-4	C8H12N2	0.00	0.00	Sweet, Fruity, Floral, Peach
2-Pyrrolidinone, 1-methyl-	872–50-4	C5H9NO	0.00	0.00	Amine
Octanoic acid, ethyl ester	106–32-1	C10H20O2	0.00	0.00	Fruity
Methane, isocyanato-	624–83-9	C2H3NO	0.00	0.00	
Aniline, *N*-methyl-	100–61-8	C7H9N	0.00	0.00	
Disulfide, dimethyl	624–92-0	C2H6S2	0.02	0.01	Garlic, Putrid, Asparagus

CON, chicken fed with standard diet; CUR, chickens fed with standard diet with 500 mg/kg CUR.

CON_ROAV and CUR_ROAV were the average means of Relative Odor Activity Method (ROAV) (n = 6), which is widely adopted to evaluate food flavor profiles.

#### 3.2.3 Analysis of key volatile flavor compounds

These distinct volatile flavor compounds accounted for the observed differences in the sensory flavor profiles of CUR and CON chicken breast muscle. The Relative Odor Activity Method (ROAV) is a widely recognized technique for evaluating food flavor profiles, and it has proven effective in detecting changes in volatile substance content in raw meat (Wang et al., 2024). Compounds with an ROAV value of ≥1 are generally considered key aroma-active compounds, signifying their substantial contribution to the overall sensory profile (Bi et al., 2022). To further elucidate the differences in the flavor compounds and profiles between CUR and CON chicken breast muscle, the ROAVs (with ROAV ≥1) of the volatile flavor compounds were calculated to quantify their contribution to the overall flavor profile (Table 3). Network graphs illustrating the relationships between flavor compounds and specific sensory flavor profiles were generated using Digraph from Flavor DB (https://cosylab.iiitd.edu.in/flavordb/). The top ten flavor profiles were sweet, green pyrazine, tetramethyl-, fruity, apple, pineapple, fatty, waxy, soapy, fresh, and nutty (Fig. 2E). This indicates that the flavor profiles of chicken breast muscle result from the synergistic interaction of various volatile flavor compounds.

2-pentylfuran was the most abundant heterocyclic volatile identified in chicken breast muscle, imparting sensory characteristics described as green bean and fresh vegetable notes. With ROAV values of 28.94 in CON and 25.33 in CUR chicken breast muscle, 2-pentylfuran originates from linoleic acid oxidation (Jiang et al., 2022). In this study, it was confirmed as a critical flavor-active compound influencing the organoleptic properties of chicken breast across all treatment groups.

Aldehydes, detected as principal volatiles in chicken (Lin et al., 2024) and other meats (Wang, Wang, et al., 2022), originate predominantly from lipid peroxidation and thermal degradation of fatty acids. Although characteristic aldehyde aromas critically define poultry's sensory appeal, CUR chicken breast exhibited reduced ROAVs of key contributors: *(E)*-2-Nonenal (100.00 vs 96.99; ROAV values of chicken breast muscle in CON vs CUR; fatty, cucumber), *(E)*-2-octenal (12.88 vs 12.43; ROAV values of chicken breast muscle in CON vs CUR; nutty, green, fatty), acetaldehyde (3.02 vs 2.55; ROAV values of chicken breast muscle in CON vs CUR; pungent, fruity, suffocating, fresh, green), and heptanal (50.85 vs 32.16; ROAV values of chicken breast muscle in CON vs CUR; citrus, fatty, rancid). Conversely, 2-methylbutanal was elevated in this study (50.85 vs 32.16; ROAV values in chicken breast muscle for CON vs CUR; cocoa, almond). Consistent with previous research (Jin et al., 2021), we confirmed curcumin's efficacy in reducing aldehyde levels, particularly *(E)*-2-nonenal and *(E)*-2-octenal in poultry muscle. Heptanal concentration significantly increased in sheep meat after 8 h of oxidation at 4 °C compared to 0 h (186.74 ± 11.21 vs 128.92 ± 8.39), signaling advanced lipid oxidation and flavor deterioration (Yang et al., 2025). As a key branched-chain aldehyde, 2-methylbutanal exhibits perceptual diversity and low odor thresholds, properties that amplify its sensory impact even when present in relatively low quantities compared to other volatiles (Chen et al., 2024). This study demonstrated that CUR supplementation promoted the accumulation of 2-methylbutanal, thereby enhancing the flavor profile of chicken breast muscle. *(E)*-2-nonenal and *(E)*-2-octenal—lipid oxidation-derived aldehydes (Sidira et al., 2015), which may be related to the antioxidant capacity of animals. Previous studies have shown an inverse relationship between an animal's antioxidant capacity and the concentration of aldehydes in its meat during early post-mortem stages (Andres et al., 2014; Andres et al., 2014; Jin et al., 2021). This finding aligns with our current results, which demonstrate that CUR supplementation significantly decreased MDA content in the chicken breast muscle.

In meat systems, ketones primarily originate from the catabolism of amino acids and the microbial β-oxidation of fatty acids (Hu et al., 2021). This study observed that CUR chicken breast displayed elevated ROAVs for key contributors such as 1-octen-3-one (Mushroom-Like) and 2-Undecanone (Orange, Fresh, Green). Conversely, 2,3-butanedione (pleasant, buttery) was reduced. Most differential ketones were significantly increased in the CUR group, exhibiting a trend opposite to that of aldehydes and MDA. MDA is a product of excessive primary lipid peroxidation and is strongly associated with the development of oxidative off-flavor. CUR acts by suppressing radical-initiated primary lipid oxidation, thereby reducing MDA accumulation and mitigating unpleasant, aldehyde-derived rancid odors. Meanwhile, lipid catabolism is redirected towards mild secondary oxidation pathways, leading to the generation of flavor-contributing methyl ketones and diketones (e.g., 2-nonanone, 2,3-octanedione). These compounds are known for their creamy, fruity, and characteristic meat-like aromas. Consequently, the increased ketone concentrations enhance the overall flavor profile of chicken breast, while the reduction in MDA effectively mitigates lipid oxidative rancidity.

Alcohols are formed through various biochemical pathways, including lipid oxidation, sugar metabolism, and the decarboxylation and dehydrogenation of amino acids, as well as the reduction of aldehydes. These compounds often possess pleasant fruity and floral aroma (Armenta et al., 2007; Moran et al., 2013; Soncin et al., 2007). Unsaturated alcohols are significant contributors to meat aroma due to their low odor detection thresholds, while saturated alcohols have minimal sensory impact given their substantially higher thresholds (Gu et al., 2013). In this study, 2-ethyl-1-hexanol (described as having rose and green notes) was identified as a key compound influencing chicken breast muscle flavor. This finding is consistent with Jin, Yang, et al. (2021), who reported the presence of the same alcohol in duck breast muscle. Furthermore, reduced concentrations of specific compounds, particularly diphenyl sulfone and heptanal characterized by citrus, fatty, and rancid notes), were found to negatively impact sensory quality when exceeding critical thresholds. These results are comparable to those of Chen et al. (2016), who observed that tea polyphenols significantly reduced flavor compounds such as hexanal and 1-octen-3-ol in enzymatic hydrolysates of surf clam, thereby improving flavor profiles.

### 3.3 Untargeted lipidomic profile

#### 3.3.1 Comparison of lipid composition of chicken breast muscle

Lipids play essential roles in the development of meat flavor development by serving as flavor precursors and generating volatile flavor compounds through oxidation and degradation. Substantial evidence confirms a strong association between that meat flavor profiles and lipid composition (Xiong et al., 2022). Hydrolyzed fatty acids, in particular, are converted into characteristic flavor compounds via thermal reactions.

**Fig. 3:**
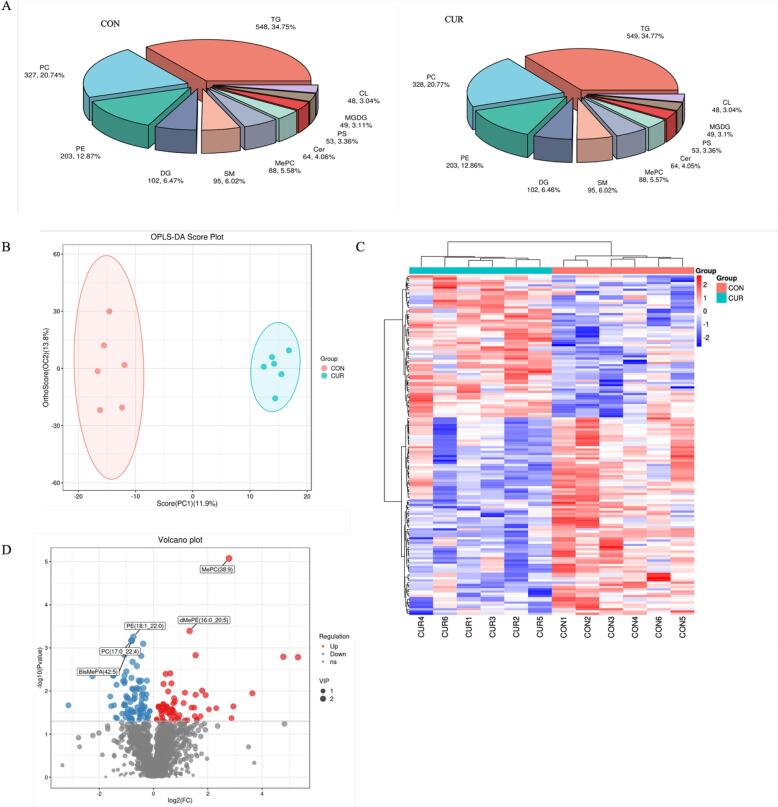
Lipid compositions analysis of the chicken breast muscle. (A) Proportion of identified lipid class classes. (B) OPLS-DA scores plot separating different chicken varieties. OPLS-DA model parameters: (R^2^Y = 0.98), (Q^2^ = 0.01); permutation validation is shown in Supplementary Fig. S2B to verify no overfitting. (C) Heat map of differentially abundance lipid classes: Red indicates higher relative content, while blue indicates lower relative content. (D) Volcano plot of differentially abundant lipids. (For interpretation of the references to colour in this figure legend, the reader is referred to the web version of this article.)

To elucidate variations in chicken flavor compounds, untargeted lipidomic profiling was performed on breast muscle samples (*n* = 6 per group) using LC-MS/MS. This analysis aimed to characterize differences in the lipid profiles between the CON and CUR groups. In total, 1977 lipid molecules were detected across the samples (Table S5). These were further classified in to 51 classes (Table S6-S7, Fig. 3A), among which the predominant classes of CUR and CON groups were triglycerides (TGs), phosphatidylcholines (PCs), phosphatidylethanolamines (PEs), diglycerides (DGs), sphingomyelins (SMs), methylated phosphatidylcholines (MePCs), phosphatidylserines (PSs), cardiolipins (CLs), ceramides (Cers) and monogalactosyl diacylglycerols (MGDGs).

Our results demonstrate that TGs, PCs and PEs are the major lipid classes in chicken breast muscle. This finding aligns with Ruan et al. (2024), who reported TGs, PCs and PEs as the predominant lipids in the breast muscle of 817 chickens, and with Zhang et al. (2024), who identified TG (39%) as the predominant lipid in laying hen breast meat. No significant difference in total TG deposition was observed between CUR (34.77%) and CON (34.75%) (*P* > 0.05). TGs, a class of neutral lipids, are crucial for various biological processes. They primarily function as the main form of lipid storage and energy reserves in organisms, and they also facilitate the transport of fatty acids. PCs and PEs are the predominant phospholipid components found in the muscle tissues. These phospholipids play critical regulatory roles in maintaining cellular morphology, modulating membrane fluidity, and influencing signal transduction pathways. Specifically, PEs are vital for the development of energy-generating organelles, while PCs are essential for brain development and lipoprotein secretion, thereby regulating lipid distribution to tissues (Wang et al., 2021). Analysis revealed that most molecular species of PCs and PEs were significantly downregulated following CUR treatment (59 decreased vs. 25 increased compared to the CON group). This indicates that CUR primarily leads to a reduction in the abundance of most PC and PE lipid species. Furthermore, chickens in the CUR group exhibited a higher concentration of VOCs in their breast muscle. This suggests that a diet supplemented with CUR alters the flavor profile of the meat. The volatile flavor of meat is substantially affected by unsaturated fatty acids, which undergo oxidation to produce a variety of volatile flavor compounds, including aldehydes, ketones, alcohols, hydrocarbons, esters, and heterocyclic compounds (Ba et al., 2013; Sanudo et al., 2000).

#### 3.3.2 Differentially lipids analysis

OPLS-DA modeling revealed distinct inter-group separations in the lipidomic profiles of the CUR and CON groups (Fig. 3B). Lower *P* values, coupled with higher VIP values, indicated were indicative of more pronounced differences in lipid abundance between the two sample groups. Screening with thresholds (VIP > 1, *P* < 0.05) identified 153 differentially abundant lipids in chicken breast muscle, the most abundant lipid classes were PCs (31), followed by TGs (21) and PEs (19) (Table S8).

Triglycerides (TGs) are ubiquitous storage lipids, playing a central role in energy homeostasis across both animal and plant kingdoms by serving as primary reservoirs of fatty acids. The enzymatic and oxidative breakdown of TGs—a process driven by lipases, lipoxygenases, and thermal degradation—produces volatile short-chain fatty acids, ketones, and aldehydes. These compounds are critical as they function as key precursors to flavor (Li et al., 2021; Mi et al., 2018). When compared to the CON group, the CUR group exhibited a higher abundance of 64 lipids and a reduction in the levels of 89 lipids. The contents of 64 lipids in the CUR group were higher than those in the CON group (*P* < 0.05), including 3 AcCas, 2 BisMePAs, 1 CLs, 3 CarEs, 4 Cer, 2 DGs, 1 Hex1Cer, 1 LBPA, 2 MGDGs, 3 MePCs, 12 PCs, 9 PEs, 2 PGs, 1 PSs, 3 SMs, 1SPHs, 12 TGs, 1 ZyE and 1 dMePE. The contents of 89 lipids in the CUR group were lower than those in the CON group (*P* < 0.05), including 3 BisMePAs, 6 CLs, 1 CerG2GNAc1, 3 Cos, 3 DGs, 17 LPCs, 6 LPEs, 4 LdMePEs, 1 MGDG, 3 MePCs, 19 PCs, 10 PEs, 2 PSs, 2 SMs and 9 TGs. The lipid of chicken breast muscle exists in the form of a large amounts of TGs, PCs and PEs.

The hydrolysis of TGs yields free fatty acids, which are crucial for the flavor of chicken breast. Conversely, the oxidation of PCs produces volatile compounds that characterize its distinct profile. A significant observation was the increased abundance of TGs in the CUR group, which is noteworthy due to the potential health benefits of TGs for humans. Furthermore, PCs are the predominant glycerophospholipid found in most animal skeletal muscle cells (Chao et al., 2020; Guo et al., 2022). They are also essential for lipoprotein secretion and regulate lipid distribution to various tissues. PE plays a vital role in the growth of energy-producing organelles. Previous research indicates that PE and PC strongly bind to free radicals, thereby effectively reducing their accumulation (Fang et al., 2022). The observed increase in TGs, PCs, and PEs in the CUR group compared to the CON group may be linked to an enhanced antioxidant capacity in chickens fed a curcumin-supplemented diet. Wang et al. (2022) showed that curcumin ameliorates liver damage and oxidative stress in rats by boosting antioxidant capacity and suppressing NLRP3 inflammasome activation.

TGs and phospholipids significantly influence meat flavor profiles due to their high content of unsaturated fatty acids. These fatty acids are prone to rapid oxidation during thermal processing and storage (Li et al., 2021). Phospholipids are more susceptible to oxidation than TGs in meat systems because of their higher unsaturated fatty acid content and increased accessibility to aqueous pro-oxidants. Phospholipids oxidation primarily targets unsaturated bonds, leading to the formation of phospholipid hydroperoxides. These unstable intermediates decompose into long-chain aldehydes and short-chain volatile carbonyls, which alter the flavor and diminish the nutritional quality of meat products. As fundamental components of cell membranes, phospholipids undergo oxidation that disrupts membrane integrity, leading to the oxidation of intracellular components and propagating oxidative cascades (Jia et al., 2021). CUR supplementation inhibited MDA accumulation in chicken breast muscle, likely by inhibiting phospholipid downregulation and consequently improving meat flavor profiles.

The heatmap generated from hierarchical clustering analysis revealed distinct segregation between the CUR and CON groups, with tight intra-group clustering of biological replicates, indicating high experimental reproducibility (Fig. 3C). To identify key differential lipids between CUR and CON supplemented chicken breast muscle, a volcano plot was generated. This analysis highlighted five predominant species: significantly upregulated MePC (38:9) and dMePE (16:0_20:5), and downregulated PE (18:1_22:0), PC (17:0_22:4), and BisMePA (42:5) in the CUR group's chicken breast muscle of compared to the CON group (Fig. 3D). Collectively, these findings demonstrate that dietary CUR substantially remodels the overall lipid profile of breast muscle. Given that these phospholipid derivatives (MePC, dMePE, and PE species) are critical endogenous precursors for lipid oxidation-derived volatile flavor substances, shifts in their abundance are hypothesized to be a significant factor influencing the final volatile flavor characteristics of chicken meat. This hypothesis was further supported by subsequent correlation analysis between differential lipids and key VOCs.

As mentioned above, CUR treatment led to a notable accumulation of methylated phospholipids, specifically MePC and dMePE, in chicken breast muscle. These phospholipids are formed through the sequential methylation of PE, a process catalyzed by PEMT (Li et al., 2023). While traditionally recognized as an enzyme primarily found in the liver, PEMT is also expressed in mammalian skeletal muscle, where it contributes to phospholipid homeostasis. Although its catalytic activity in muscle is comparatively lower than in the liver, genetic studies in mice have demonstrated that skeletal PEMT effectively regulates PE metabolism and the levels of methylated phospholipids (Verkerke et al., 2019). Given CUR's potent antioxidant properties, it is plausible that CUR modulates PEMT activity, thereby promoting the synthesis of MePC and dMePE from PE. This alteration could consequently impact the precursors available for lipid oxidation and, ultimately, the development of meat flavor.

### 3.4 Correlation analysis of volatile flavor compounds and lipid compounds

**Fig. 4:**
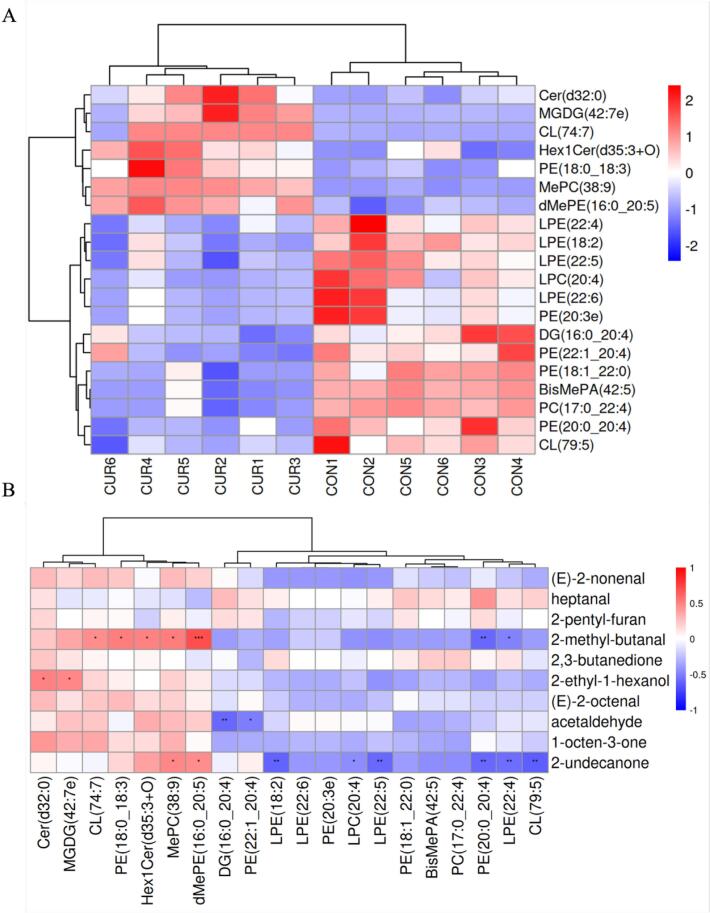
(A) Hierarchical clustering heatmap of the top 20 differentially abundant lipids (highest VIP) between CON and CUR chicken breast muscle. (B) Pearson correlation heatmap of differential lipids and key volatile flavor compounds. All correlation analyses were subjected to FDR multiple testing correction prior to visualization. Black dots mark correlation pairs showing statistical significance after FDR correction; cells without black dots correspond to non-significant correlations after correction, which are reserved only for raw data exhibition and not analyzed in this study. * and ** represent *P* < 0.05 and *P* < 0.01, respectively.

Significant flavor profile differences were observed between CUR and CON. Notably, volatile flavor compounds including *(E)*-2-nonenal, heptanal, 2-pentylfuran, 2-methyl-butanal, 2,3-butanedione, 2-ethyl-1-hexanol, *(E)*-2-octenal, acetaldehyde, 2-undecanone, and 1-octen-3-one were identified as key flavor contributors (ROAV >1, *P* < 0.05) in both groups. With the exception of 2-ethyl-1-hexanol, 2-methylbutanal, 1-octen-3-one, and 2-undecanone, all other identified flavor compounds exhibited lower concentrations in CUR chicken breast muscle compared to CON. To further investigate the relationship between flavor variations, the top 20 differential lipids were selected for correlation analysis, as detailed in Table S8. Fig. 4A illustrates the VIP-ranked hierarchical clustering of these top 20 lipids, which displayed significantly different abundances between the CUR and CON groups.

Fig. 4B visualizes the correlation between major volatile flavor compounds and differential lipids, utilizing FDR-adjusted correlation data. Only statistically significant correlations are presented in the revised heatmap. The results indicate a strong positive correlation between 2-methyl-butanal and various lipids (CL (74:7), PE (18:0_18:3), Hex1Cer(d35:3 + O), MePC (38:9) and dMePE (16:0_20:5)), and negatively corrected with PE (20:0_20:4) and LPE (18:2). 2-ethyl-1-hexanol was strongly associated with Cer (d32:0) and MGDG (42:7e). Acetaldehyde was negatively corrected with DG (16:0_20:4) and PE (22:1_20:4). 2-undecanone was positively correlated with MePC (38:9) and dMePE (16:0_20:5), and negatively corrected with multiple lipids (LPE (18:2), LPC (20:4), LPE (22:5), PE (20:0_20:4), LPE (22:4) and CL (79:5)). Significant correlations were observed between these volatile aldehydes, ketones and differential lipids. Flavor formation is governed by complex metabolic pathways, necessitating further mechanistic experiments, such as enzyme activity detection, to elucidate the rules of precursor conversion.

## 4 Conclusion

This study utilized multi-omics techniques to investigate differences in flavor precursors and VOCs in chicken breast muscle from CON and CUR groups, alongside measurements of MDA and IMF content. Dietary CUR supplementation significantly inhibited lipid oxidation, consequently reducing the formation of diphenyl sulfone and heptanal (associated with citrus, fatty, and rancid odors). Interestingly, no significant difference in IMF content was observed in the chicken breast muscle. Marked distinctions were found in the VOCs and lipid profiles between the CUR and CON groups. The flavor profiles of chicken breast muscle exhibited considerable variation, encompassing attributes such as sweet, green/pyrazine (tetramethyl-), fruity (apple, pineapple), fatty, waxy, soapy, fresh, and nutty. Based on the VOCs analysis, curcumin supplementation demonstrably improved the flavor of chicken breast muscle by suppressing off-flavors derived from diphenyl sulfone and heptanal. Correlation analysis revealed strong relationships between 2-methyl-butanal, 2-ethyl-1-hexanol, 2-undecanone, and acetaldehyde were strongly correlated with various lipids, indicating their susceptibility to lipid content. This study offers a comprehensive analysis of VOCs and lipid characteristics in chicken breast muscle from both CUR and CON groups, providing valuable insights for enhancing meat flavor, inhibiting the accumulation of undesirable compounds such as diphenyl sulfone and heptanal, and developing flavor-improved meat products. Sajid and Siddique (2024) previously demonstrated that natural agricultural by-products can function as dietary fiber sources to produce high-quality meat products with satisfactory flavor and sensory acceptance, further supporting the utility of natural additives in developing flavor-improved meat products. Moreover, Mehwish et al. (2024) underscored the growing importance of nanotechnology in advanced food packaging, which can further preserve meat freshness, stabilize flavor compounds, and extend shelf life. Therefore, future research will concentrate on three specific areas: optimizing the curcumin supplementation dosage, integrating systematic sensory evaluation with enzyme and lipoxygenase activity detection to elucidate the regulatory mechanism of CUR on lipid and volatile compound formation, and combining natural antioxidant addition with novel packaging technologies to develop high-quality, stable chicken products.

## CRediT authorship contribution statement

**Sanjun Jin:** Writing – original draft, Visualization, Project administration, Methodology, Investigation, Funding acquisition, Data curation. **Kaige Yang:** Writing – review & editing, Methodology, Investigation. **Gaofeng Pan:** Writing – review & editing, Project administration, Data curation, Conceptualization. **Ping Wang:** Writing – review & editing. **Chaoqi Liu:** Writing – review & editing, Conceptualization. **Xinxin Li:** Writing – review & editing, Resources, Investigation. **Qingqiang Yin:** Writing – review & editing, Project administration, Conceptualization. **Juan Chang:** Writing – review & editing, Validation, Software, Funding acquisition. **Lijun Wang:** Writing – review & editing, Visualization, Validation, Conceptualization. **Fushan Lu:** Writing – review & editing, Project administration.

## Declaration of competing interest

The authors declare that they have no known competing financial interests or personal relationships that could have appeared to influence the work reported in this paper.

## Data Availability

Data will be made available on request.
