# Supplementary figures and images for: A lipidomic and flavoromic approach to map the lipid profile and related volatile flavor compounds in the fresh breast meat of chickens fed curcumin
Source: Food Chem X. 2026 Jun 30;37:104156. doi: 10.1016/j.fochx.2026.104156 (PMC13355225; doi:10.1016/j.fochx.2026.104156)

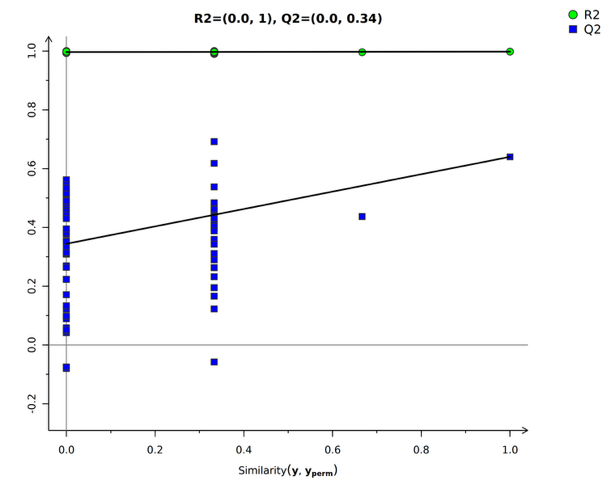


Supplementary Fig.S1


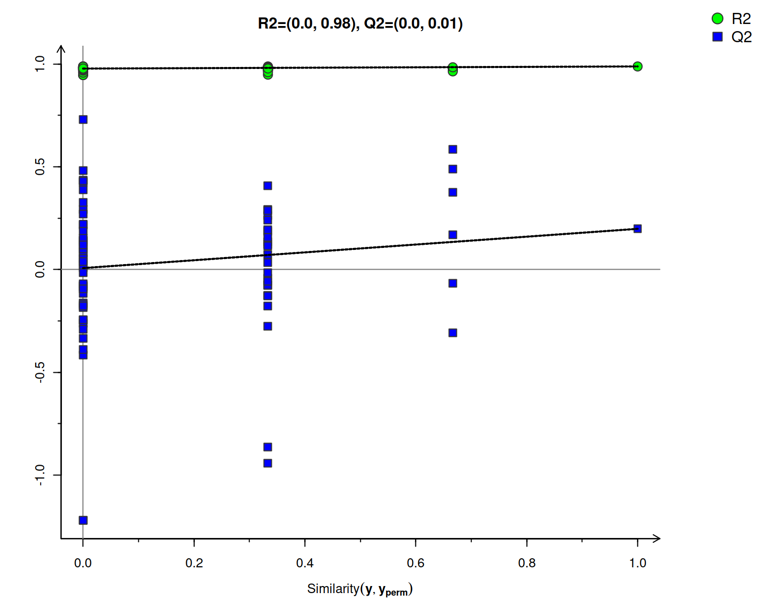


Supplementary Fig.S2

Supplement: Supplementary file 1 — Supplementary material 1 [file mmc1.docx]
