# Supplementary material for: A lipidomic and flavoromic approach to map the lipid profile and related volatile flavor compounds in the fresh breast meat of chickens fed curcumin
Source: Food Chem X. 2026 Jun 30;37:104156. doi: 10.1016/j.fochx.2026.104156 (PMC13355225; doi:10.1016/j.fochx.2026.104156)
Supplement: Supplementary file 2 — Supplementary material 2 [file mmc2.docx]

Table S1 Composition and nutritional levels of experimental diet (%, air-dried basis)

| Ingredients（%） | 1-21 d | 22-42 d |
| --- | --- | --- |
| Corn | 62.43 | 67.40 |
| Soybean meal | 30.00 | 25.24 |
| fish meal | 2.00 | 1.00 |
| Soybean oil | 2.00 | 3.00 |
| CaHPO_4_ | 1.50 | 1.40 |
| Limestone | 1.23 | 1.18 |
| Met | 0.10 | 0.11 |
| Lys | 0.07 |  |
| NaCl | 0.37 | 0.37 |
| Premix^1^ | 0.30 | 0.30 |
| Total | 100.00 | 100.00 |
| Nutrient components |  |  |
| ME（MJ/kg）^2^ | 12.43 | 12.86 |
| CP（%） | 20.02 | 17.96 |
| Ca（%） | 0.98 | 0.88 |
| TP（%） | 0.68 | 0.62 |
| AP（%）^2^ | 0.41 | 0.36 |
| Met（%）^2^ | 0.42 | 0.40 |
| Lys（%）^2^ | 1.10 | 0.89 |

^1^ Premix provides: 12000 IU of vitamin A per kilogram of feed; Vitamin D_3_ 3000 IU; Vitamin E 20 IU; Vitamin K_3_ 1.0 mg; Vitamin B_1_ 2.0 mg; Vitamin B_2_ 6.0 mg; Vitamin B_6_ 3.5 mg; Vitamin B_12_ 0.01 mg; D-biotin 0.15 mg; Folic acid 1.25 mg; Nicotinic acid 35 mg; D-Calcium pantothenate 10 mg; Copper 8.0 mg; Iron 100 mg; Manganese 80 mg; Zinc 60 mg; Iodine 0.45 mg; Selenium 0.35 mg.
